# Effects of oral adenosine-5′-triphosphate supplementation on athletic performance, skeletal muscle hypertrophy and recovery in resistance-trained men

**DOI:** 10.1186/1743-7075-10-57

**Published:** 2013-09-22

**Authors:** Jacob M Wilson, Jordan M Joy, Ryan P Lowery, Michael D Roberts, Christopher M Lockwood, Anssi H Manninen, John C Fuller, Eduardo O De Souza, Shawn M Baier, Stephanie MC Wilson, John A Rathmacher

**Affiliations:** 1 Department of Health Sciences and Human Performance, The University of Tampa, Tampa FL, USA; 2 Department of Biomedical Sciences, College of Veterinary Medicine, University of Missouri, Columbia, MO, USA; 3 AP Nutrition, LLC, Missoula MT, USA; 4 Metabolia Oulu, Oulu, Finland; 5 Metabolic Technologies Inc., Iowa State University Research Park, Ames, IA, USA; 6 Laboratory of Neuromuscular Adaptations to Strength Training, School of Physical Education and Sport, University of São Paulo, São Paulo, Brazil; 7 Department of Nutrition, IMG Academy, Bradenton, FL, USA; 8 Department of Animal Science, Iowa State University, Ames, IA, USA

**Keywords:** Adenosine triphosphate, Exercise performance, Power, Strength, Muscle hypertrophy, Sports nutrition

## Abstract

**Background:**

Currently, there is a lack of studies examining the effects of adenosine-5′-triphosphate (ATP) supplementation utilizing a long-term, periodized resistance-training program (RT) in resistance-trained populations. Therefore, we investigated the effects of 12 weeks of 400 mg per day of oral ATP on muscular adaptations in trained individuals. We also sought to determine the effects of ATP on muscle protein breakdown, cortisol, and performance during an overreaching cycle.

**Methods:**

The study was a 3-phase randomized, double-blind, and placebo- and diet-controlled intervention. Phase 1 was a periodized resistance-training program. Phase 2 consisted of a two week overreaching cycle in which volume and frequency were increased followed by a 2-week taper (Phase 3). Muscle mass, strength, and power were examined at weeks 0, 4, 8, and 12 to assess the chronic effects of ATP; assessment performance variables also occurred at the end of weeks 9 and 10, corresponding to the mid and endpoints of the overreaching cycle.

**Results:**

There were time (*p* < 0.001), and group x time effects for increased total body strength (+55.3 ± 6.0 kg ATP vs. + 22.4 ± 7.1 kg placebo, *p* < 0.001); increased vertical jump power (+ 796 ± 75 ATP vs. 614 ± 52 watts placebo, *p* < 0.001); and greater ultrasound determined muscle thickness (+4.9 ± 1.0 ATP vs. (2.5 ± 0.6 mm placebo, *p* < 0.02) with ATP supplementation. During the overreaching cycle, there were group x time effects for strength and power, which decreased to a greater extent in the placebo group. Protein breakdown was also lower in the ATP group.

**Conclusions:**

Our results suggest oral ATP supplementation may enhance muscular adaptations following 12-weeks of resistance training, and prevent decrements in performance following overreaching. No statistically or clinically significant changes in blood chemistry or hematology were observed.

**Trial registration:**

ClinicalTrials.gov NCT01508338

## Introduction

Adenosine-5′-triphosphate’s (ATP) role as the primary intracellular energy source for body tissues is well established [1]. In addition, ATP also has extensive extracellular functions that are primarily mediated through purinergic (P2Y and P2X) membrane receptors ubiquitously present in many cell types [2]. One extracellular-mediated function of ATP includes the modification of muscle excitability (i.e., increasing skeletal muscle calcium permeability and blocking chloride efflux) and vasodilation [3,4]. Moreover as a co-transmitter, ATP operates on both the central and peripheral nervous systems to elicit local tissue modifications during neurotransmission [5].

It has been reported that the half-life of infused ATP is less than one second [6-8], ATP is rapidly taken up and stored by erythrocytes [6]. This rapid uptake by erythrocytes is central to its role in affecting blood flow and oxygen delivery to oxygen-depleted tissue [9]. Specifically, there is a tight coupling between oxygen demand in skeletal muscle and increases in blood flow. Erythrocytes regulate this response by acting as “oxygen sensors” [10]. When oxygen is low in a working muscle region, the red blood cell deforms, and releases ATP [10,11]. The result is vasodilation and greater blood flow to the working musculature, thereby enhancing nutrient and oxygen delivery [10,11]. Long-term oral administration of ATP has been shown to increase both the uptake and synthesis of ATP in the erythrocytes of rodents [12]. Collectively, these findings suggest that oral ATP supplementation may elicit ergogenic outcomes on skeletal muscle without elevating plasma ATP concentrations.

Research by Jordan et al. [13] demonstrated that 225 mg per day of enteric-coated ATP supplementation for 15 days resulted in increased total bench press lifting volume (i.e. sets•repetitions•load [kg]) as well as within-group set-one repetitions to failure. More recently, Rathmacher et al. [14] found that 15 days of 400 mg per day of ATP supplementation increased minimum peak torque for the final two sets of a dynamometer test. The increases suggest that orally delivered ATP may reduce muscle fatigue and enable a higher force output during repeated high-intensity bouts of exercise. These aforementioned findings lead us to hypothesize that dietary supplementation with ATP may be beneficial to both the exercising and less active muscle tissue.

The novelty of ATP as an oral supplement has limited data available in regards to its chronic effects in humans. To date, no studies have examined the chronic effects of oral ATP supplementation on body composition or indicators of athletic performance when combined with a periodized resistance training (RT) protocol. However, provided that short-term supplementation in the absence of a training intervention has resulted in positive outcomes in muscle performance, it is plausible to suggest that ATP may have long-term ergogenic effects in periodized RT regimens. Therefore, the primary purpose of this study was to test the hypothesis that supplementation with oral ATP would improve measures of power, strength, and skeletal muscle mass during a 12-week RT protocol. The second purpose was to assess the safety of the supplement over 12 weeks through blood chemistry and hematology measures.

## Methods

### Study design

This was a randomized, double-blind, placebo- and diet-controlled, parallel groups with repeated measures study design. Both groups were assigned to a 12-week periodized RT protocol. Blinding occurred via an outside researcher who sent the supplement and placebo in identical opaque capsules. This researcher was not involved in direct data collection, and did not meet any of the subjects. For this reason neither the researchers conducting the study nor the subjects knew who was in each group. Moreover the code was not broken until after all of the data were entered into Microsoft® Excel, and sent to an outside researcher who was also blinded to the treatment groups. The protocol was divided into three phases. Phase one consisted of a three times per week non-linear periodized RT program for weeks 1–8, modified from Kraemer et al. [15]. Phase two consisted of a two-week overreaching cycle during weeks 9 and 10. Finally, phase three consisted of participants tapering for weeks 11 and 12. Muscle mass and body composition were measured at baseline and at the end of weeks 4, 8, and 12. Muscle strength, vertical jump power, Wingate peak power (PP), creatine kinase (CK), C-reactive protein (CRP), free and total testosterone, and perceived recovery were measured at baseline and after weeks 4, 8, 9, 10 and 12. Additionally, CK, CRP, free and total testosterone, and perceived recovery were measured after week 1 of training. Protein breakdown was assessed using urinary 3-methylhistidine: Creatinine ratio (3-MH:Cr) at baseline and after weeks 1 and during the overreaching phase at weeks 8, 9, and 10. These variables were used to assess the effects of ATP on performance, hormone status, and indices of muscle damage and recovery during an overreaching cycle. The ClinicalTrials.gov registration ID was NCT01508338.

### Participants

Twenty-four resistance-trained males were selected for the study. However, due to injury three subjects dropped out of the study leaving 21 subjects (11 ATP supplemented and 10 placebo supplemented) aged 23.4 ± 0.7 years, with an average one-repetition maximum (1RM) squat, bench press, and deadlift of 1.71 ± 0.04, 1.34 ± 0.03 and 2.05 ± 0.04 times their bodyweight were recruited for the study. Participants could not participate if they were taking an anti-inflammatory agent, a performance-enhancing supplement, if they smoked, or if they had consumed nutritional supplements during the three months prior to data collection. Each participant signed an informed consent approved by the University of Tampa Institutional Review Board before participating in the study.

### Muscle strength, power, body composition and skeletal muscle hypertrophy testing

After familiarization procedures, muscle strength was assessed via 1RM testing of the back squat, bench press, and deadlift. Each lift was performed as described by the International Powerlifting Federation rules [16]. We used an intraclass correlation coefficient (ICC) (2,k) formula [17] to determine the reliability of repeated measures within testers. The ICC for strength measures ranged from 0.96 to 0.98. Body composition (lean body mass, fat mass, and total mass) was determined by dual-energy x-ray absorptiometry (DXA; Lunar Prodigy enCORE 2008, Madison, Wisconsin, U.S.A.). Skeletal muscle hypertrophy was determined via the combined changes in ultrasonography-determined muscle thickness of the vastus lateralis (VL) and vastus intermedius (VI) muscles. The mean of three measurements by the same blinded investigator were taken at 50% of femur length over the mid-belly of the muscle with the subjects lying in a supine position. The precision for the test-retest of muscle thickness measurements was 0.975.

Muscle power was assessed during maximal cycling and jumping movements. During the cycling test, volunteers were instructed to cycle against a predetermined resistance (7.5% of body weight) as fast as possible for 10 seconds [18]. The saddle height was adjusted to the individual’s height to produce a 5–10° knee flexion while the foot was in the low position of the central void. A standardized verbal stimulus was provided to each participant. Power output was recorded in real time during the 10-second sprint test, by a computer connected to the standard cycle ergometer (Monark model 894e, Vansbro, Sweden). Peak power (PP) was recorded using Monark Anaerobic Wingate Software, Version 1.0 (Monark, Vansbro, Sweden). The ICC of muscle peak power was 0.96.

Measurements of PP were also taken during a vertical jump (VJ) test performed on a multicomponent AMTI force platform (Advanced Mechanical Technology, Inc., Watertown, MA), interfaced with a personal computer at a sampling rate of 1000 Hz [19]. Data acquisition software (LabVIEW, version 7.1; National Instruments Corporation, Austin, TX) was used to calculate PP. Peak power was calculated as the peak combination of ground reaction force and peak velocity during the accelerated launch on the platform. The ICC of VJ power was 0.97.

### Supplementation and diet control

Prior to the study, participants were randomly assigned to receive either 400 mg per day of ATP disodium or maltodextrin (placebo), consumed orally via a two-piece gelatin capsule 30 minutes prior to RT sessions. On non-training days, participants were instructed to consume one dose on an empty stomach prior to breakfast. The placebo and ATP capsules (400 mg) were obtained directly from the commercial manufacturer (TSI USA Inc., Missoula, MT) and were produced in compliance with U.S. cGMPs for dietary supplements. The Certificate of Analysis for both the placebo and ATP capsules were provided and verified the supplement contents. Moreover, we verified the capsule’s purity by HPLC. Two weeks prior to and throughout the study, participants were placed on a diet consisting of 25% protein, 50% carbohydrates, and 25% fat by a registered dietician who specialized in sport nutrition. Participants met as a group with the dietitian, and they were given individual meal plans two weeks prior to the onset of the study. Diet counseling was continued on an individual basis throughout the study.

### Resting blood draws

All blood draws were obtained after an overnight 12-hour fast via venipuncture by a trained phlebotomist, and were scheduled at the same time of day to avoid influences of hormonal variations. Whole blood was collected and transferred into appropriate tubes and centrifuged at 1500 x g for 15 min at 4°C. Resulting serum and plasma were then aliquoted and stored at −80°C until subsequent analyses. A portion of the blood samples taken at weeks 0, 4, 8, and 12 were used for measurements (ANY LAB TEST NOW®, Tampa, Fl) of glucose, blood urea nitrogen, creatinine, eGFR, Na, K, Cl, CO_2_, Ca, protein, albumin, globulin, albumin:globulin ratio, total bilirubin, alkaline phosphatase, aspartate aminotransferase, and alanine aminotransferase. A complete blood count was also performed on each blood sample. The commercial laboratory performing the safety analysis was instructed to inform investigators of any abnormal values in any parameters measured during the study time points measured. In the case of an abnormal value, the subject would be told and not be allowed to continue the study. Because no abnormal values were detected during the study in these healthy and highly fit subjects, the total aggregate numbers were not presented to the investigators until the end of the study.

### Biochemical analysis

Samples were thawed once and analyzed in duplicate for each analyte. Serum free and total testosterone, cortisol, and C-reactive protein (CRP) were assayed via ELISA kits obtained from Diagnostic Systems Laboratories (Webster, TX). All hormones were measured within the same assay and on the same day to avoid compounded inter-assay variance. Intra-assay variance was less than 3% for all analytes. Serum creatine kinase (CK) was measured using colorimetric procedures at 340 nm (Diagnostics Chemicals, Oxford, CT).

### Perceived recovery status scale

Perceived Recovery Status (PRS) Scale was measured at all measurement times and in particular at weeks 8, 9, and 10 to assess participant recovery during the overreaching phase. The PRS Scale consists of values between 0–10, with 0–2 being very poorly recovered and characterized by anticipated declines in performance; 4–6 is defined as low-to-moderately recovered and characterized by no expected change in performance; and, 8–10 represents high perceived recovery and correlates strongly with increases in performance [20].

### Statistics

A one-way ANOVA model was used to analyze the baseline characteristic data using the Proc GLM procedure in SAS® (SAS Institute, Cary, NC)_._ The main effect of treatment (Trt) was included in the model. Actual values for muscle strength and power, body composition, muscle damage, hormonal status, and PRS changes over the 12-week study were analyzed by using a repeated measures ANOVA using the Proc Mixed procedure in SAS®. The initial baseline value (e.g. week 0) was used as a covariate with the main effects of Time, Trt, and the interaction Trt*Time in the model. The overreaching cycle of the study was further assessed by using repeated measures ANOVAs with the Proc Mixed procedure in SAS®. Values measured at the week-8 time point were used as a covariate with the main effects of Time, Trt, and Trt*Time for the overreaching phase (phase two). The Least Squares Means procedure was used to compare Trt means at each time point. Statistical significance was determined at *p* ≤ 0.05.

## Results

### Participant characteristics

There were no differences in age (placebo = 23.0 ± 1.2, ATP = 23.7 ± 0.9 yrs), height ( placebo = 180.6 ± 2.3, ATP = 179.0 ± 1.0 cm), or body mass (placebo = 87.4 ± 4.3, ATP = 85.7 ± 1.7 between the treatments at the start of the study.

### Muscle strength and power

**Figure 1:**
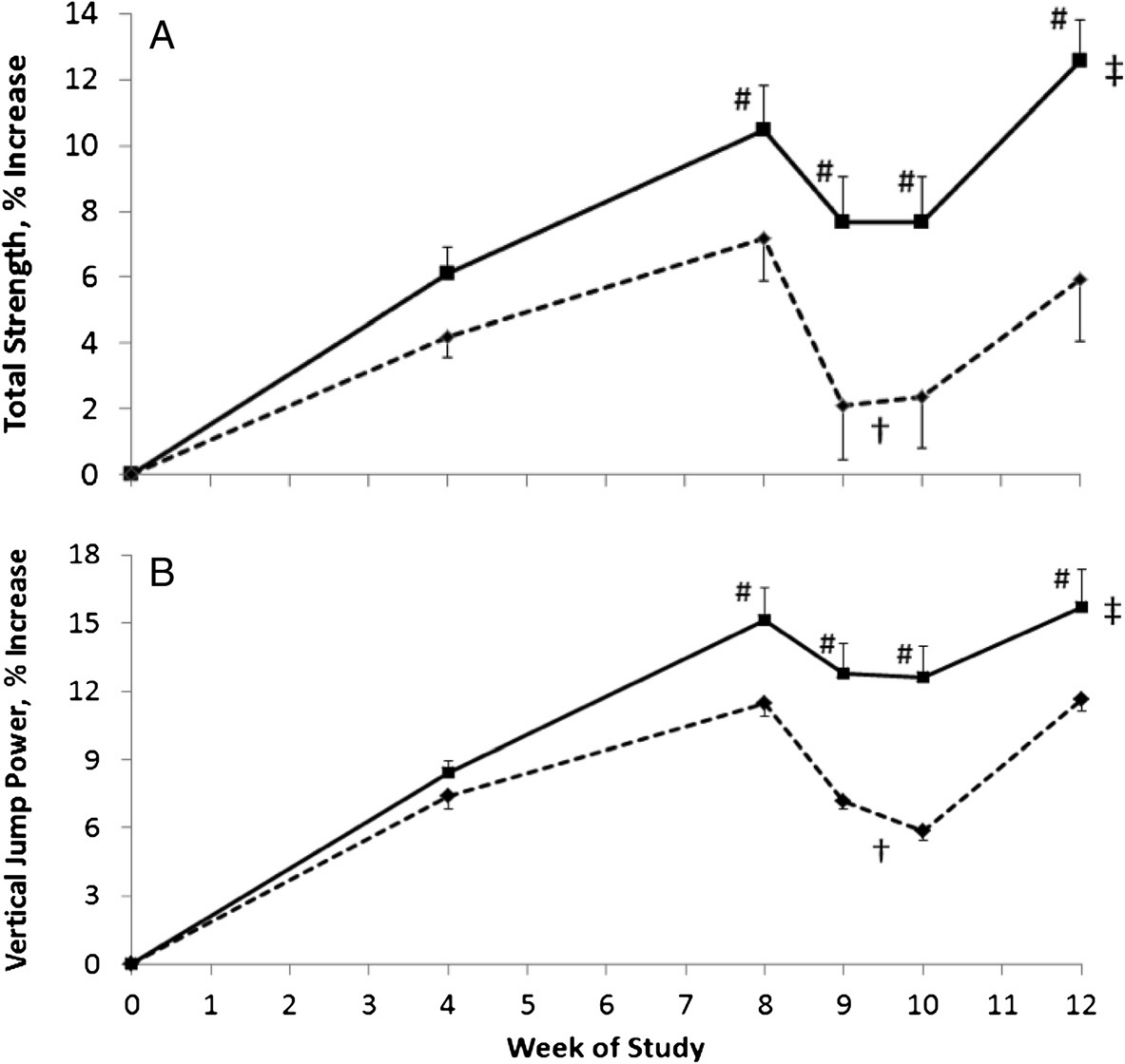
**Effects of oral ATP supplementation on total strength and vertical jump power. (A)** Percent change in total strength as the of sum of the 1-RM in bench press, squat, and deadlift in male participants undergoing 12 weeks of intense, periodized resistance training with an overreaching cycle during weeks 9 and 10. Participants were supplemented with either a Placebo - - ♦- - (n=10) or 400 mg ATP/d —∎— (n=11) during the 12-week training program. When compared with placebo supplementation total strength was increased with ATP supplementation over the 12 week study (‡ p < 0.001, Trt*time). During the overreaching cycle of the study, total strength decreased in the placebo-supplemented group relative to the ATP-supplemented group during weeks 9 and 10 († *p* < 0.001, Trt*time). Additionally, participants supplemented with ATP had greater mean strength at the 8, 9, 10, and 12 week time-points compared with placebo-supplemented participants (# p < 0.05). **(B)** Percent change in vertical jump power in male participants undergoing 12 weeks of intense, periodized resistance training with an overreaching cycle during weeks 9 and 10. Participants were supplemented with either a Placebo - - ♦- - (n=10) or 400 mg ATP /d —∎— (n=11) during the training program. ATP supplementation resulted in a greater increase in vertical jump power over the 12 week study (‡ *p* < 0.001, Trt*time). The increased intensity of the overreaching cycle during the study resulted in a significant decrease in vertical jump power in the placebo-supplemented participants compared with the ATP-supplemented participants during weeks 9 and 10 († *p* < 0.007). Additionally, ATP-supplemented participants had greater vertical jump power at 8, 9, 10, and 12 weeks of study compared with placebo-supplemented participants (# *p* < 0.05).

Both groups increased their muscle strength (Table 1, Figure 1A, *t*-test, *p* < 0.001). However, supplementation with ATP resulted in significantly greater increases in the 1RM for the squat, deadlift, and total strength compared with placebo over the 12-week study (Trt*time, *p* < 0.001, 0.002, and 0.001, respectively). The ATP supplementation resulted in strength increases of 12.9% and 16.4% for squat and deadlift, respectively. However, in the placebo group, RT alone resulted in increases of 4.4 and 8.5% for squat and deadlift, respectively. The total strength increases over the 12-week study were 5.9% (22.4 ± 7.1 kg) in the placebo-supplemented participants and 12.6% (55.3 ± 6.0 kg) in the ATP-supplemented participants (Figure 1A, Trt*time, *p* < 0.001). Mean total strength in response to ATP supplementation was greater at 8, 9, 10, and 12 weeks than mean total strength in the placebo-supplemented participants (*t*-test, *p* < 0.05). During phase 2, the overreaching cycle, the placebo-supplemented group had a 22.6 ± 5.1 kg decrease in total strength, while the ATP-supplemented group decreased only 12.0 ± 2.5 kg in total strength during the same test period (Table 1, Figure 1A, Trt, *p* < 0.007).

**Table 1:** **Effect of ATP supplementation on muscle strength and power in participants performing a 12 week weight training regimen.**^**a**^

	**Week of Study**						
	**0**	**4**	**8**	**9**	**10**	**12**	***p*-value**^**b**^
Squat, kg							
Placebo	145.2 ± 10.2	151.6 ± 11.0	156.4 ± 11.6	150.7 ± 10.4	147.2 ± 10.7	151.3 ± 10.3	
ATP	146.1 ± 5.8	155.2 ± 7.7	160.7 ± 7.6	159.0 ± 6.1^#^	158.6 ± 6.9^#^	165.3 ± 8.3^#^	<0.001
Bench Press, kg							
Placebo	115.2 ± 9.4	119.3 ± 9.4	121..4 ± 9.3	113.4 ± 7.2	115.7 ± 8.0	119.3 ± 8.7	
ATP	117.2 ± 5.5	122.1 ± 5.0	124.5 ± 5.2	120.0 ± 4.8	121.2 ± 5.5	124.2 ± 5.4	0.65
Deadlift, kg							
Placebo	169.8 ± 11.0	177.0 ± 10.7	182.5 ± 10.7	173.2 ± 10.8	174.8 ± 8.1	182.0 ± 9.2	
ATP	176.2 ± 4.9	189.2 ± 5.9	200.3 ± 7.4^#^	193.4 ± 6.4^#^	193.8 ± 8.3^#^	205.2 ± 6.9^#^	0.002
Total Strength, kg							
Placebo	430.2 ± 28.1	448.0 ± 28.9	460.2 ± 29.6	437.3 ± 26.1	437.6 ± 25.1	452.6 ± 25.9	
ATP	439.4 ± 14.9	466.5 ± 17.1	485.6 ± 18.1^#^	472.4 ± 15.2^#^	473.6 ± 18.3^#^	494.7 ± 17.9^#^	<0.001
Wingate Peak Power, Watts							
Placebo	882.2 ± 54.1	930.3 ± 55.7	985.3 ± 64.7	922.9 ± 61.9	939.9 ± 65.7	977.4 ± 66.5	
ATP	882.8 ± 27.0	960.9 ± 36.1	1003.5 ± 40.4	964.2 ± 38.0	974.7 ± 39.8	1011.9 ± 42.4	0.48
Vertical Jump power, Watts							
Placebo	5318 ± 410	5711 ± 439	5918 ± 443	5697 ± 436	5626 ± 431	5932 ± 452	
ATP	5185 ± 203	5618 ± 213	5954 ± 202^#^	5838 ± 218^#^	5827 ± 208^#^	5981 ± 207^#^	<0.001

^a^Mean ± SEM for n = 11 ATP (400 mg ATP/d taken 30 min prior to exercise) and n = 10 placebo supplemented participants.

^b^Probability of treatment by time difference between the placebo and the ATP treatments over the 12-week study. The mixed model ANOVA in SAS® was used with the main effects of treatment, time and treatment by time, with the value for week 0 used as a covariate.

^#^Significantly different than corresponding placebo, *t*-test (*p* < 0.05).

Muscle power measured by Wingate PP and vertical jump power increased pre-to-post-test similarly in both groups during the RT protocol (Table 1 and Figure 1B; *t*-test, *p* < 0.001). However, ATP supplementation significantly increased vertical jump power when compared to placebo (Table 1 and Figure 1B; Trt*time *p* < 0.001). The ATP-supplemented participants had a 15.7% (796 ± 75 watts) increase in vertical jump power compared with an 11.6% (614 ± 52 watts) increase in placebo-supplemented participants over the 12-week period. During phase 2, the overreaching cycle, the placebo-supplemented participants had a 5.0% (−291 ± 25 watts) decrease in power from week 8 to week 10, while the ATP-supplemented participants had a 2.2% decrease (−126 ± 19 watts; Trt*time, *p* < 0.001).

### Body composition and muscle hypertrophy measurement

After 12 weeks of RT, lean body mass (LBM) increased within both groups (Table 2, *t*-test, *p* < 0.001), whereas fat percentage was decreased with training only in the ATP-supplemented participants (Table 2*t*-test, *p* <0.01). The ATP supplementation did not affect overall body weight or fat percentage during the study, but it did significantly increase LBM gain during the 12-week training period (Trt*time, *p* < 0.009). The ATP-supplemented participants gained 4.0 ± 0.4 kg of LBM whereas the placebo-supplemented participants gained 2.1 ± 0.5 kg of LBM. The quadriceps muscle thickness increased after 12 weeks of RT (Table 2, Time, *p* < 0.001). Additionally, ATP supplementation resulted in a 9.4% (4.9 ± 1.0 mm) increase in quadriceps muscle thickness compared with a 4.9% (2.5 ± 0.6 mm) increase in response to placebo (Table 2, Trt*time *p* < 0.02).

**Table 2:** **Effect of ATP supplementation on body composition and Quadriceps thickness in participants performing a 12 week weight training regimen.**^**a**^

	**Week of Study**				
	**0**	**4**	**8**	**12**	***p*-value**^**b**^
Weight, kg					
Placebo	87.4 ± 4.3	88.3 ± 4.6	88.7 ± 4.8	87.7 ± 4.7	
ATP	85.7 ± 1.7	86.9 ± 2.0	87.0 ± 2.0	87.0 ± 2.1	0.49
DXA LBM, kg					
Placebo	68.5 ± 2.6	70.0 ± 2.3	71.2 ± 2.4	70.5 ± 2.4	
ATP	67.7 ± 2.0	70.1 ± 1.9	71.4 ± 2.0^#^	71.7 ± 1.9^#^	.009
DXA Fat,%					
Placebo	21.0 ± 1.1	19.8 ± 1.6	18.6 ± 1.9	18.6 ± 1.7	
ATP	19.5 ± 1.8	18.1 ± 1.8	16.6 ± 1.6	16.0 ± 1.5	0.76
Quad, mm					
Placebo	50.2 ± 2.1	52.2 ± 2.3	52.6 ± 2.4	52.7 ± 2.4	
ATP	50.9 ± 0.9	53.4 ± 1.3	54.8 ± 1.7	55.8 ± 1.8^#^	0.02

^a^Mean ± SEM for n = 11 ATP (400 mg ATP/d taken 30 min prior to exercise) and n = 10 placebo supplemented participants.

^b^Probability of treatment by time difference between the placebo and the ATP treatments over the 12-week study. The mixed model ANOVA in SAS® was used with the main effects of treatment, time and treatment by time, with the value for week 0 used as a covariate.

^#^Significantly different than corresponding placebo, *t*-test (*p* < 0.05).

### Muscle damage, hormonal status and performance recovery scale

Muscle damage assessed by blood CK was affected by training (Table 3, Time, *p* < 0.001). Supplementation with ATP was unable to attenuate the increase in CK at either the initiation of training (weeks 0 to 1) or during phase 2, the overreaching cycle (weeks 9 and 10), where the sudden change in training volumes caused blood CK to increase. Muscle protein degradation was measured by the urinary 3-MH:Cr ratio (Table 3) during the initiation of training (weeks 0 to 1) and overreaching cycle (weeks 9 and 10). During week 8 of the study, ATP-supplemented subjects had a higher urinary 3-MH:Cr ratio compared to the placebo group (*t*-test, *p* < 0.05). However, the 8-week values were not different than baseline for both treatment groups. The results indicate that when training volume increased during weeks 9 and 10, ATP supplementation significantly decreased muscle protein degradation compared with placebo (Trt*time, *p* < 0.007). During phase 2, the overreaching cycle, protein degradation increased 23.7 ± 4.5% in the placebo-supplemented, but not ATP supplemented participants. Supplementation with ATP did not affect changes in CRP, cortisol, or free or total testosterone levels when compared with placebo supplementation (Table 3).

**Table 3:** **Blood creatine kinase (CK), urinary 3-methylhistidine (3MH), C-reactive protein (CRP), cortisol, free and total testosterone, and perceived recovery score (PRS) in participants performing a 12 week weight training regimen and supplemented with either a placebo or ATP.**^**a**^

		**Week of Study**						
	**0**	**1**	**4**	**8**	**9**	**10**	**12**	***p*-value**^**b**^
CK, IU/L								
Placebo	141 ± 12	582 ± 77	373 ± 13	246 ± 29	484 ± 52	528 ± 72	187 ± 21	
ATP	145 ± 8	500 ± 71	324 ± 14	234 ± 32	426 ± 44	449 ± 62	160 ± 20	0.91
24 h 3MH:Cr, μmol:mg								
Placebo	0.127 ± 0.007	0.130 ± 0.003	Nm^c^	0.123 ± 0.004	0.134 ± 0.005	0.152 ± 0.005	Nm	
ATP	0.136 ± 0.008	0.127 ± 0.007	Nm	0.143 ± 0.007^#^	0.143 ± 0.008	0.131 ± 0.012^#^	Nm	0.007
CRP, mg/L								
Placebo	1.9 ± 0.7	1.1 ± 0.1	1.3 ± 0.3	2.0 ± 0.7	1.6 ± 0.7	1.2 ± 0.2	1.6 ± 0.4	
ATP	1.4 ± 0.4	1.1 ± 0.1	1.2 ± 0.2	1.9 ± 0.6	1.7 ± 0.6	1.1 ± 0.1	1.2 ± 0.2	0.99
Cortisol, μg/dL								
Placebo	19.7 ± 1.1	20.8 ± 1.3	19.0 ± 1.2	19.2 ± 0.4	22.0 ± 0.4	23.6 ± 0.3	20.3 ± 0.6	
ATP	20.9 ± 1.2	20.5 ± 1.3	18.4 ± 1.4	19.0 ± 0.4	21.5 ± 0.4	22.6 ± 0.2	19.7 ± 0.6	0.86
Free Testosterone, ng/dL								
Placebo	103 ± 13	112 ± 10	119 ± 6	111 ± 9	98 ± 6	100 ± 9	113 ± 12	
ATP	112 ± 13	114 ± 9	118 ± 6	117 ± 11	108 ± 7	110 ± 10	125 ± 13	0.93
Total Testosterone, ng/dL								
Placebo	591 ± 73	620 ± 58	625 ± 55	585 ± 58	551 ± 46	536 ± 88	605 ± 72	
ATP	660 ± 67	645 ± 54	695 ± 60	645 ± 60	621 ± 49	592 ± 84	673 ± 69	0.83
PRS^d^								
Placebo	9.1 ± 0.3	4.7 ± 0.4	7.0 ± 0.3	7.6 ± 0.2	4.8 ± 0.3	4.4 ± 0.3	7.6 ± 0.2	
ATP	9.6 ± 0.2	4.9 ± 0.4	7.5 ± 0.3	8.2 ± 0.3	5.5 ± 0.4	5.5 ± 0.4	8.6 ± 0.4	0.61

^a^Mean ± SEM for n = 11 ATP (400 mg ATP/d taken 30 min prior to exercise) and n = 10 placebo supplemented participants.

^b^Probability of treatment by time difference between the placebo and the ATP treatments over the 12-week study. The mixed model in SAS® was used with the main effects of treatment, week and treatment by week, with the value for week 0 used as a covariate. The mixed model in SAS® was also used for 3MH:Cr: however, 3MH:Cr was measured only at weeks 0, 1, 8, 9, and 10. The model used these values with week 0 as a covariate for determining the overall treatment by time effect for 3MH:Cr.

^c^Not measured.

^d^Perceived recovery score is rated on the participants feeling of recovery from the last workout on a scale of 0-10. 0-2 indicates poor recovery and an anticipated decline in performance; 4-6 indicates low to moderate recovery and expected similar performance; and 8-10 indicates highly perceived recovery and expected increases in performance.

^#^Significantly different than corresponding placebo, *t*-test (*p* < 0.05).

Muscle recovery and readiness to train in the next training session was measured by PRS score (Table 3). While no overall 12-week Trt*time effect of ATP supplementation was observed, the effects of ATP supplementation compared with placebo at weeks 10 and 12 may indicate that ATP supplementation improved perceived recovery (Table 3).

### Safety assessment using blood chemistry and hematology

Blood chemistry and hematology analyses were performed at baseline (week 0) and at weeks 4, 8, and 12 of the study; the data are shown in Tables 4 and 5, which displays blood chemistry and hematology values, respectively. No statistically or clinically significant changes in blood chemistry or hematology were observed over the 12-week ATP supplementation period when compared with placebo supplementation. There were no adverse events reported in this study.

**Table 4:** Blood chemistry values

	**Placebo**					**ATP**					
	Before^*^	**SEM**^**†**^	**After**	**SEM**	**% Change**	**Before**	**SEM**	**After**	**SEM**	**% Change**	***p- *Value**^**‡**^
Glucose, mmol/l	4.48	0.08	4.51	0.09	0.6	4.62	0.07	4.65	0.04	0.7	1.00
Uric acid, mmol/l	0.29	0.02	0.29	0.02	1.0	0.29	0.02	0.29	0.01	1.3	0.99
Blood Urea Nitrogen, mmol/l	5.61	0.26	5.75	0.30	2.6	5.63	0.18	5.67	0.21	0.6	0.99
Creatinine, μmol/l	87.0	2.4	89.1	4.2	2.5	86.2	2.3	85.0	4.0	−1.5	0.80
eGRF, ml/min	101	2.2	101	3.6	0.6	105	3.7	107	3.7	1.7	0.93
Sodium, mmol/l	140	0.6	140	0.4	0.1	139	0.5	140	0.5	0.5	0.64
Potassium, mmol/l	4.3	0.1	4.3	0.1	−0.2	4.4	0.1	4.4	0.1	0.0	0.68
Chloride, mmol/l	101	0.4	101	0.4	−0.1	100	0.5	101	0.5	0.5	0.31
CO_2_, mmol/l	24.8	0.6	25.1	0.4	1.2	24.6	0.3	24.2	0.4	−1.5	0.77
Calcium, mmol/l	2.40	0.03	2.39	0.03	−0.2	2.44	0.02	2.43	0.02	−0.5	0.98
Protein, g/l	72.0	1.1	72.2	1.5	0.3	72.5	1.0	72.7	1.0	0.2	0.98
Albumin, g/l	46.6	0.5	46.0	0.8	−1.3	46.7	0.7	46.3	0.7	−0.8	0.53
Globulin, g/l	26.0	0.6	26.1	0.7	0.4	24.6	0.6	24.6	0.4	0.0	0.97
A:G Ratio	1.80	0.05	1.77	0.06	−1.6	1.91	0.06	1.89	0.05	−1.1	0.57
Total Bilirubin, μmol/l	13.4	2.5	12.9	2.6	−3.8	9.8	1.3	9.6	1.3	−1.7	0.71
Alkaline Phosphatase, IU/l	77.1	3.2	82.1	3.6	6.5	80.2	4.4	79.7	4.5	−0.6	0.16
Aspartate Aminotransferase, IU/l	25.0	1.9	24.0	2.0	−4.0	24.8	1.5	24.9	1.4	0.4	0.49
Alanine Aminotransferase, IU/l	26.6	2.8	26.6	2.8	0.0	22.1	2.0	22.3	1.9	0.8	0.90

^*^Values before and after 12 weeks of either placebo or ATP (400 mg/d) administration.

^†^Standard error of the mean.

^‡^*p-* value for Trt*time effect, the model included data from weeks 4 and 8 (not shown).

**Table 5:** Blood hematology values

	**Placebo**					**ATP**					
	Before^*^	**SEM**^**†**^	**After**	**SEM**	**% Change**	**Before**	**SEM**	**After**	**SEM**	**% Change**	***p *Value**^**‡**^
WBC, x10^9^/l	5.93	0.38	5.98	0.34	0.8	6.08	0.24	6.07	0.22	−0.1	0.67
RBC, x10^12^/l	5.16	0.10	5.19	0.09	0.7	5.13	0.04	5.07	0.05	−1.1	0.30
Hemoglobin, g/l	156	2.8	155	2.4	−1.0	157	2.2	156	2.0	−0.8	0.97
Hematocrit, l/l	0.46	0.01	0.46	0.01	0.0	0.47	0.01	0.46	0.01	−1.8	0.54
MCV, μm^3^	89.7	0.8	89.3	1.0	−0.5	90.6	0.8	89.7	0.8	−1.0	0.94
MCH, pg/cell	30.5	0.40	30.2	0.33	−1.1	30.4	0.32	30.5	0.44	0.3	0.42
MCHC, g/l	339	3.1	336	2.5	−0.9	334	3.9	340	4.4	2.1	0.16
RDW,%	13.4	0.1	13.4	0.1	0.0	13.1	0.1	13.2	0.1	0.8	0.62
Platelets, x10^9^/l	247	9.0	236	9.1	−4.2	248	9.5	217	5.1	−12.5	0.64
Neutrophils,%	46.5	3.1	47.4	2.5	1.9	47.6	3.0	47.8	2.0	0.5	0.80
Lymphocytes,%	39.6	3.2	38.4	2.6	−3.0	39.0	2.8	38.8	1.7	−0.6	0.74
Monocytes,%	10.3	0.6	9.4	0.7	−8.7	9.3	0.5	9.6	0.6	3.0	0.57
Eosinophils,%	3.6	0.5	3.6	0.5	0.0	3.4	0.4	3.4	0.4	0.0	0.98
Basophils,%	0.6	0.2	0.7	0.2	16.7	0.4	0.2	0.4	0.2	−0.3	0.83
Neutrophils, x10^9^/l	3.2	0.6	3.3	0.5	2.5	3.0	0.3	2.9	0.2	−2.3	0.27
Lymphocytes, x10^9^/l	2.4	0.2	2.5	0.3	4.1	2.4	0.2	2.4	0.1	2.9	0.99
Monocytes, x10^9^/l	0.67	0.08	0.62	0.08	−7.5	0.82	0.20	0.61	0.05	−25.6	0.62
Eosinophils, x10^9^/l	0.21	0.02	0.21	0.02	0.0	0.21	0.03	0.22	0.03	4.4	0.32
Basophils, x10^9^/l	0.02	0.01	0.01	0.01	−50.0	0.00	0.00	0.00	0.00	0.0	0.41

^*^Values Before and After 12 weeks of either placebo or ATP (400 mg/d) administration.

^†^Standard error of the mean.

^‡^*P* value for Trt*time effect, the model included data from weeks 4 and 8 (not shown).

## Discussion

The primary findings of this study were that individuals consuming 400 mg of oral ATP daily demonstrated ergogenic effects with ATP supplementation, since they showed greater gains in muscle mass, lean body mass, strength and power when compared to a placebo-matched control.

### The effects of ATP on skeletal muscle strength and power development

Strength and power are two of the most critical attributes underlying success in athletics [21,22]. These variables are intimately related and allow athletes to be successful in their respective sport [23,24]. Prior to our research, there was limited data evaluating the effects of supplemental ATP on physiological responses that would improve long-term muscular performance. For example, Jordan et al. [13] demonstrated that 14 days of orally ingested ATP positively influenced exercise performance. Specifically, they demonstrated that 225 mg per day of ATP for 14 days resulted in within-group increases in set-one total repetitions performed and bench press total training volume. More recently, Rathmacher et al. [14] found that 400 mg of supplemental ATP per day for 15 days was effective in improving set-two leg muscle minimum peak torque and tended to decrease set-three leg muscle fatigue during three successive sets of knee extension exercises. Interestingly, the changes observed during the aforementioned studies occurred despite final ATP supplementation being provided to test participants 3 [13] and 12 hours [14] prior to post-testing data collection. The prolonged lag time between supplementation and testing may explain the inconclusive, albeit within-group significant effects observed by Jordan et al. [13] and the somewhat minimal but between group effects on torque and fatigue observed by Rathmacher et al. [14] Furthermore, since the 400 mg per day dose used by Rathmacher et al. [14] was divided into two doses throughout the day, the effective pre-exercise testing dose consumed prior to the 12-hour supplement withdrawal would have only been 200 mg of ATP.

The current investigation employed once per day supplementation of a 400 mg dose of ATP where on training and testing days participants consumed the supplement 30 minutes prior to exercise. We found the ATP supplementation resulted in strength increases for the squat and deadlift of 12.9% and 16.4%, respectively, when compared to RT alone in the placebo (4.4% and 8.5%). In comparison to the two former studies, it is likely that the more robust changes observed within the current investigation are the result of total ATP dose and dose timing relative to testing procedures.

With regard to muscle power, we found that vertical jump peak power was more responsive to ATP supplementation (+15.3%) as compared to placebo (+11.5%). Rathmacher et al. [14] have speculated that supplemental ATP may provide cumulative benefits in strenuous, repetitive, and exhaustive exercise activities, which could lead to improvements in muscle responses. However, we have to point out that the mechanisms that ATP mediates changes in skeletal muscle performance are still under investigation and need to be fully elucidated. It can be speculated that these functional changes are a factor of small, transient increases in extracellular ATP and its metabolites. For example, Sandona et al. [25] presented evidence that [ATP]_ex_ increases skeletal muscle Ca^2+^ influx and the release of Ca^2+^ from the sarcoplasmic reticulum, thereby affecting muscle contractile properties. Specifically, increasing skeletal muscle Ca^2+^ influx and intracellular concentrations have been shown to significantly increase both the total number of thin filaments binding and the speed at which the filaments slide [26]. These aforementioned findings provide a clue as to how the ATP supplementation might modulate the increases in muscle strength and power. However, these speculations must be eventually validated with direct research.

### The effects of ATP on skeletal muscle mass and changes in lean body mass

To our knowledge, this study represents the first formal investigation of the effects of oral ATP supplementation on lean body mass and muscle thickness following a chronic RT program. Our results indicated greater increases in LBM and muscle thickness in response to oral ATP versus placebo. We can speculate on a number of possible reasons for the ergogenic effects of ATP on muscle mass we have observed. In addition to ATP’s capacity to buffer fatigue during repeated high volume sets and increase total training volume, the supplement may increase skeletal muscle blood flow, thereby enhancing muscle O_2_ recovery. This is critical as muscle deoxygenation is associated with decreased performance under repeated high intensity contractions [27]. Specifically, extracellular ATP directly promotes the increased synthesis and release of nitric oxide (NO) and prostacyclin (PGl_2_) within skeletal muscle and therefore directly affects tissue vasodilation and blood flow [28]. This is supported by research suggesting increased vasodilation and blood flow in response to intra-arterial infusion [29] and exogenous administration of ATP. It can be speculated that these changes in blood flow may lead to an increased substrate pool for skeletal muscle by virtue of increased glucose and O_2_ uptake [9]. If this is the case the resulting outcome would be an improved recovery response via a greater energetic environment for anabolic processes capable of supporting exercise-induced changes in LBM and hypertrophy. However, these are currently only suggested possible mechanisms and need to be verified in future research.

### The effects of ATP on recovery from high intensity training

Overtraining and overreaching are two of the most complicated occurrences in sport. The primary tools utilized to detect overtraining and overreaching include changes in serum indices of skeletal muscle damage [30], anabolic and catabolic hormone status [31], perceived recovery [20], and muscle protein breakdown. However, the consensus seems to dictate that the number one indicator of overreaching and overtraining are short- and long-term decrements in performance, respectively [32]. The cause of overreaching appears to be an imbalance between training stimulus and recovery. If the stimulus exceeds the athlete’s adaptive capacity, decrements in performance will result; ultimately taking weeks (overreaching) to months (overtraining) to fully recover. For ethical reasons, a great deal of research in strength and conditioning has centered on overreaching protocols versus overtraining [32].

The present study attempted to overreach participants through increasing training frequency and volume. Our results indicated that the overreaching cycle was able to decrease muscle power and strength, and overreaching increased protein breakdown. However, these effects were blunted in the ATP group. It is interesting to note that protein breakdown was not blunted during phase 1 (weeks 1–8) by ATP supplementation. In fact, although protein breakdown in the ATP supplemented group was not significantly different than baseline, it appeared slightly higher at week 8 relative to the control. We can speculate that under normal conditions of training, when glycogen levels are likely adequate those participants supplementing with ATP were able to maintain higher intensities, which would result in higher rates of protein breakdown. However, when exposed to greater training frequencies, glycogen levels are likely to be depleted, thus preventing higher intensities from being performed. As such ATP supplementation is able to blunt a rise in protein breakdown relative to a placebo group. Thus, it appears that oral ATP may be able to increase fatigue resistance during a two-week intensive, high-volume overreaching protocol. Following a two-week taper in which volume was reduced, the placebo group regained their baseline performance while the ATP group experienced increases in both muscle strength and power. These results indicate that a typical overreaching stimulus overwhelms recovery capabilities in a non-supplemented state. However, the ability for ATP to speed recovery may provide athletes with a novel method to promote positive training adaptations.

While at first look these results may appear to be only pertinent to athletic performance, it is important to understand that several non-sport activities place people at risk for deterioration of performance in life threatening situations where performance is critical. A primary example includes combat athletes / military personal who are often times placed in extreme overreaching and overtraining environments that may take months to recover from [33]. These are often times highly conditioned individuals whose lives and mission may depend on the prevention of decay in strength, and power.

### Effects of chronic ATP supplementation on safety parameters

The results of this study suggest that 400 mg of oral ATP administered daily had no effect on hemoglobin, white blood cells, blood glucose, liver, or kidney function. These results are in agreement with Coolen et al. [34] who reported that up to 5000 mg per day, during 28 days, of oral ATP resulted in no significant changes in any blood or urine measured safety parameters. Therefore, it appears likely that doses of 400 mg/day of oral ATP for up to 12 weeks can be considered safe and presented no clinically relevant adverse effects.

### Bioavailability of oral ATP supplementation

Recently, Coolen et al. questioned the bioavailability of oral ATP [2]. However, the biological pool where ATP is measured will determine the results of bioavailability analysis. If sampled in venous portal blood, oral ATP is indeed bioavailable [13]. Coolen et al. did not analyse venous portal blood. If sampling systemic plasma or whole blood, oral ATP will have very low bioavailability even in very high doses. Paradoxically, Kichenin & Seman observed that repeated administration of oral ATP led to progressive diminution of plasma ATP [12]. Nevertheless, their study demonstrated that oral ATP supplementation can indeed produce biological responses. Over all, due to the nature of the ATP molecule, its bioavailability is quite difficult to determine. While our results suggest that oral ATP supplementation can significantly impact athletic performance, skeletal muscle hypertrophy and recovery, the current study did not utilize methodologies to investigate the potential mechanism(s) for the ergogenic effects we observed.

### Limitations

This study, similar to others, has limitations. This study included all male subjects; therefore, future research will need to expand our study design to a female population. We also acknowledge that that these results occurred with a highly trained population under extreme training conditions. Chronic studies will need to be conducted in untrained individuals under normal training loads. Finally, we did not assess changes in muscular O_2_ concentrations or changes in intramuscular ATP. We suggest that future researchers conduct these studies using near infrared spectroscopy to access intramuscular O_2_ concentrations and magnetic resonance spectroscopy to assess any changes in ATP charge. Without these measures, caution should be taken when interpreting the actual mechanisms of action of the supplement.

## Conclusions

The collective findings of our current study suggest that oral supplementation with ATP in combination with high intensity, periodized RT, increases muscle mass, strength, and power compared with a placebo-matched control. Moreover, when faced with greater training frequencies, oral ATP may prevent typical declines in performance that are characteristic of overreaching. Future research should seek to elucidate the underlying mechanisms through which ATP operates to promote improvements in training adaptations.

## Abbreviations

ATP: Adenosine-5′-triphosphate; Ck: Creatine kinase; cGMP: Current Good Manufacturing Practices; DXA: Dual-energy x-ray absorptiometry; LBM: Lean body mass; PP: Peak power; PGl2: Prostacyclin; RT: Resistance training.

## Competing interests

JMW, JMJ, RPL, MDR, EOD, SMCW and AHM declare no competing interests. JR, JF, and SB are employed by Metabolic Technologies, Inc. which engages in business trade with TSI (USA), Inc. CL was a consultant of TSI, Inc.

## Authors’ contribution

JMW, RPL, JMJ were involved in study design, training subjects, biochemical analysis of blood, data interpretation, and manuscript preparation. JAR, SMB, and JCF were involved in study design, supplement preparation, analysis of 3-MH, statistical analysis, and manuscript preparation. MDR, CML, AHM, and EOD were critical for study design, and manuscript preparation. SMCW served as the study’s sports dietitian and also assisted in study design. All authors read and approved the final manuscript.
